# The VvWRKY37 Regulates Bud Break in Grape Vine Through ABA-Mediated Signaling Pathways

**DOI:** 10.3389/fpls.2022.929892

**Published:** 2022-06-16

**Authors:** Feng-Pan Wang, Pan-Pan Zhao, Lei Zhang, Heng Zhai, Muhammad Abid, Yuan-Peng Du

**Affiliations:** ^1^Lushan Botanical Garden, Chinese Academy of Sciences, Jiujiang, China; ^2^National Key Laboratory of Crop Biology, National Research Center for Apple Engineering and Technology, College of Horticulture Science and Engineering, Shandong Agricultural University, Tai’an, China; ^3^Guangdong Provincial Key Laboratory for Plant Epigenetics, College of Life Sciences and Oceanography, Shenzhen University, Shenzhen, China; ^4^College of Biological and Enology Engineering, Taishan University, Tai’an, China

**Keywords:** grapevine, bud break, WRKY, abscisic acid, transcriptional regulation

## Abstract

Dormancy is a common survival strategy in plants to temporarily suspend visible growth under unsuitable conditions. The elaborate mechanism underlying bud break in perennial woody plants is gradually illustrated. Here, we identified a grape vine WRKY transcription factor, *VvWRKY37*, which was highly expressed in dormant buds. It was particularly induced by the application of exogenous abscisic acid, and depressed on exposure to gibberellin and low temperature (4°C) stress at the transcript level. The yeast one-hybrid assay confirmed that VvWRKY37 had a transcriptional activity. Ectopic over-expression of *VvWRKY37* significantly delayed bud break of transgenic poplar plants. As an ABA-inducible gene, *VvWRKY37* also depressed the expression of ABA catabolic gene *CYP707A*s and enhanced the accumulation of endogenous ABA in transgenic poplar plants. The molecular pieces of evidence showed that VvWRKY37 preferentially recognized and bound W-box 5′-G/CATTGACT/C/G-3′ *cis*-element *in vitro*. Additionally, VvABI5 and VvABF2 acted as the upstream transcriptional activators of *VvWRKY37 via* protein-DNA interactions. Taken together, our findings provided valuable insights into a new regulatory mechanism of WRKY TF by which it modulates bud break through ABA-mediated signaling pathways.

## Introduction

The growth and development of sessile plants is typically affected by climatic changes. Consequently, plants have evolved regulatory mechanisms to thrive well under unsuitable environmental conditions, i.e., freezing temperature. Perennial plants have developed a survival strategy that enables them to suspend and resume growth activities under cyclic changes in the environment. Meristem dormancy is a common strategy in most temperate perennial plants for their survival, development, and morphogenesis. Vegetative or floral buds of perennial plants bear pivotal responsibilities for growth and reproduction after dormancy release and thus ensure the sustainability of the plants after enduring unfavorable growth conditions. After dormancy establishment, endodormancy requires sufficient chilling intensity or other stimuli treatment (such as hydrogen cyanamide and sodium azide) before transitioning to ecodormancy (Xin and Browse, 2010). Ecodormancy can restore growth after a shift to growth-inductive conditions, indicating bud break. The signals originating from developmental (i.e., phytohormones), physiological (i.e., water, sugar, and phytochrome), and environmental (i.e., day length and temperature) factors play essential roles in the complex crosstalk regulating the bud dormancy and bud break (Chmielewski et al., 2017; Singh et al., 2017; Liu and Sherif, 2019).

So far, bud dormancy at the physiological level is well-studied (Parada et al., 2016; Chmielewski et al., 2017; Singh et al., 2017; Rubio et al., 2019), but the molecular and genetic mechanisms of the signaling networks that regulate dormancy and bud break are obscure. Previously, researchers have made numerous attempts to dissect genetic regulatory mechanisms underlying bud dormancy and break (Yordanov et al., 2014; Busov et al., 2016; Singh et al., 2018; Tylewicz et al., 2018; Artlip et al., 2019; Yang et al., 2019). Short-day length and low temperature synergistically promoted bud endodormancy and abscisic acid (ABA) accumulation in plants (Singh et al., 2017; Liu and Sherif, 2019). The ABA content gradually increased during bud dormancy establishment and decreased during endodormancy (Chmielewski et al., 2017). The overaccumulation of ABA prevented precocious growth during bud dormancy by blocking growth-related intracellular signaling cascades (Tylewicz et al., 2018). The ABA deficient mutant plants exhibited impaired dormancy potential (Singh et al., 2018), whereas ABA sufficient mutant and transgenic lines showed enhanced dormancy phenotypes (Yang et al., 2020). The ABA levels in winter buds increased in autumn and reached to a maximum level during endodormancy, and then persistently declined after endodormancy in grape plants (Rubio et al., 2019). The grape vines overexpressing the ABA catabolic gene *VvA8H-CYP707A4* gene enhanced ABA catabolism and bud break (Zheng et al., 2018a). All in all, an association between ABA content and bud behavior strengthens the argument for the regulatory role of ABA in bud dormancy induction, duration, and release.

Several studies have revealed the regulatory role of dormancy-related transcriptional factors (TFs), including dormancy-associated MADS-box (DAM) in aspen (Singh et al., 2018), pear (Yang et al., 2020), kiwifruit (Wu et al., 2017); APETALA2/Ethylene responsive factor in poplar (Yordanov et al., 2014), grape, and apple (Busov et al., 2016), Chinese cherry (Zhu et al., 2021); C-repeat binding factor (CBF/DREB) in peach (Artlip et al., 2019), pear (Li et al., 2019), bZIP-like TFs (ABFs) in pear (Yang et al., 2020), grape (Zheng et al., 2015); and WRKY TFs in peach (Chen et al., 2016). Interestingly, the above-mentioned dormancy-responsive TFs are directly or indirectly involved in dormancy-related phytohormone signal transduction cascades (Liu and Sherif, 2019). The expression patterns of WRKY TFs were correlated with bud dormancy in fruit trees (Chen et al., 2016; Vimont et al., 2019). Additionally, previous significant advances in ABA signaling studies have suggested that WRKY TF plays an essential role in ABA-responsive signaling networks (Rushton et al., 2012). In *Arabidopsis*, WRKY33 impeded ABA accumulation *via* transcriptional repression of the ABA biosynthesis genes *NCED3* and *NCED5* and activating the ABA catabolic gene *CYP707A3* in pathogen-induced defense response (Liu et al., 2015). It is reported that WRKY41 directly targets *ABI3* to trigger ABA signaling, which is beneficial for seed dormancy (Ding et al., 2014). Besides, WRKY6 positively regulated ABA-mediated seed germination by directly downregulating *RAV1* gene expression (Huang et al., 2016). OsWRKY29 represses seed dormancy in rice caused by the inhibitory effect of ABA signaling (Zhou et al., 2020).

Here, a grape WRKY TF (*VvWRKY37)* was characterized for its regulatory role in dormant winter bud. Moreover, we showed that the transcript abundance of *VvWRKY37* was induced by exogenous application of ABA and repressed on exposure to exogenous application of gibberellin (GA) and cold temperature. It is widely accepted that phytohormone ABA and cold temperatures are associated with plant dormancy in perennial plants. The present study provided evidence that VvWRKY37 is involved in bud dormancy and break *via* an ABA-mediated signaling pathway. This study also uncovered a VvWRKY37-mediated regulatory network by which GA and/or low-temperature exposure probably played respective roles in regulating bud dormancy and break.

## Materials and Methods

### Plant Materials and Treatments

The self-rooted grape cultivar “Cabernet Sauvignon” (*Vitis vinifera*) from a vineyard located in Tai’an city, Shandong province, China (N36°09′49.24″, E117°08′53.73″) was used in this study. All berries were sampled from various 5-year-old grape vines cultivated using a vertical trellis system at the following stages: 7 days post-anthesis (dpa), (pepper-corn size berry), 35 dpa (pre-veraison berry), 70 dpa (veraison berry), and 105 dpa (harvest-ripe berry). Each berry was immediately peeled and deseeded. The skin, pulp, and seeds were frozen in liquid nitrogen and stored at –80°C except for the fruit at 7 dpa because of their small berries, making it difficult to separate the various tissues. The bud dormancy phenotyping was done for various dormancy-related parameters under environmental conditions. The dormancy depth was measured by using a previously described method (Pérez et al., 2007). The time period for the phase transition of endodormancy to ecodormancy was determined from December 4, 2015 to January 5, 2016 (Supplementary Figure 1). The appearance of visible green tissue at the tip of the winter bud was indicative of bud break. The data for bud break was collected on April 2, 2016 in spring. The winter buds were collected at the following phases: leaf fall in autumn (October 15, 2015), endodormancy (November 24, 2015), phase transition from endodormancy to ecodormancy (December 24, 2015), ecodormancy (February 15, 2016), and bud break in spring (April 2, 2016). The scales were quickly removed from the winter buds before freezing in liquid nitrogen.

Detached canes with more than 10 buds were sampled from a vineyard at the beginning of leaf fall. The experiment was repeated three times, and each replication contains 10 canes. The bottoms of canes were soaked in the following solution: ABA (10 μM), GA_3_ (5 μM), and mock solution (ethanol, 0.06%), respectively. The canes were placed in a growth chamber set at 25°C/20°C temperature and 16 h/8 h (light/dark) photoperiod. For low-temperature treatment, the bottoms of the canes were soaked in sterile water. The scales were carefully removed from each bud, and then the canes were transferred to low-temperature conditions (4°C) or normal conditions (20°C) as control. More than 20 buds were sampled for each time point. The scales were quickly removed from the winter buds before freezing in liquid nitrogen. Total RNA was extracted for analyzing transcript abundance analysis.

The poplar cultivar “84K” (*Populus alba* × *P. glandulosa*) was used as a wild genotype. *In vitro*-grown poplar shoots were sub-cultured every month using a maintenance medium, and the normal MS medium (20 g/L sucrose, 7. g/L agar powder, pH = 5.8) was supplemented with phytohormone NAA at 0.1 mg/L. The newly cultured shoots were grown at 25°C/20°C under a 16 h/8 h (light/dark) photoperiod in transparent glass bottles.

### Plasmid Constructions and Plant Transformations

Standard molecular biological techniques and homologous recombination technology (Vazyme, Nanjing, China) were employed for gene cloning and plasmid construction. The coding sequence of *VvWRKY37* without stop codon was cloned from the first-strand cDNA, generating an in-frame C-terminal fusion to the GFP gene downstream of CaMV 35S promoter (*35S*:*VvWRKY37-GFP*). The recombinant plasmid was introduced into the LBA4404 strain of *Agrobacterium tumefaciens*.

The 1,165 bp upstream promoter fragment of *VvWRKY37* was amplified using grape vine (Cabernet Sauvignon) genomic DNA. The DNA fragment was inserted into the pCAMBIA1300-GN vector to generate the *pVvWRKY37*:*GUS* construct. Finally, the construct was introduced into the GV3101 strain of *Agrobacterium tumefaciens* and transformed into wild-type *A. thaliana* plants *via* the agrobacterium-mediated floral dip method. The T_0_ seedlings were surface sterilized and screened on selective media. The well-grown resistant seedlings were transplanted and identified *via* genotyping using gene-specific primer pairs. The T_3_ homozygous transgenic lines were used for subsequent experiments.

Leaves from *in vitro*-grown poplar “84K” shoot were used for transgenic plant regeneration. Fully expended young leaves were collected and co-cultivated with *Agrobacterium tumefaciens* strain LBA4404 harboring *35S*:*VvWRKY37-GFP* vector. The leaves were kept under dark conditions for 2 days and transferred into a regeneration medium containing 50 mg/L kanamycin and 250 mg/L cefotaxime. The regenerated shoots with a minimum of two expended leaves were cut and transferred into a rooting medium (kanamycin, 50 mg/L; cefotaxime, 250 mg/L; and NAA, 0.1 mg/L). Well-rooted plantlets were transplanted in plastic pots (10.5-cm height × 10.5-cm diameter) filled with a 50% peat and 50% perlite mixture. The pots were then covered with plastic bags for 1 week and incubated under constant growth conditions [25°C/20°C and a 16-h/8-h (light/dark) photoperiod].

### Total RNA Extraction, RT-qPCR, and Semi-Quantitative RT-PCR

Plant total RNA was extracted using the CTAB solution [2% cetyltrimethyl ammonium bromide, 2.5% polyvinyl pyrrolidone K30, 2-M NaCl, 100-mM Tris–HCl, 25-mM EDTA-Na_2_, 0.05% spermidine, 2% β-mercaptoethanol (added just before use)] as described previously with slight modifications (Gambino et al., 2008), or TRIzol™ Reagent (Thermo Fisher, Waltham, United States) by following the manufacturer’s instructions. Microspectrophotometer NanoDrop One (Thermo Fisher, Waltham, United States) was used to quantify the total RNA, and the RNA integrity was confirmed using 1% agarose gel electrophoresis. The first-strand cDNA was synthesized from 1 μg of total RNA by PrimeScript™ RT Reagent Kit with genome DNA Eraser by following the manufacturer’s protocol (TaKaRa, Tokyo, Japan). The stock solution of the first-stand cDNA was diluted 20 to 40 times with sterilized ddH_2_O for RT-qPCR analysis. Then, RT-qPCR was carried out in a 96-well plate using Bio-Rad CFX96 (Bio-rad, Hercules, United States). Each well contained a total volume of 14-μl reaction mixtures consisting of 1 μl of cDNA, 7 μl of *TransStart*^®^ Top Green qPCR SuperMix (TransGen, Beijing, China), 0.5 μl of each primer (10 mM), and 5 μl of sterilized ddH_2_O. The PCR amplification procedure was as follows: initial denaturation at 94°C for 30 s, followed by 40 cycles at 94°C for 5 s, and 60°C for 30 s. Dissociation melting curve analysis was used to verify the PCR product specificity. Three housekeeping genes, *Vvactin*, *AtGAPDH*, and *PtUbi*, were used as internal references for grape, *Arabidopsis*, and poplar, respectively. The expression levels for genes were calculated by using the 2^–ΔΔCt^ method. The gene-specific primer pairs were synthesized using GENEWIZ (Suzhou, China), and the sequences for primer pairs are given in Supplementary Table 1.

### Yeast One-Hybrid Assay

A previously described method was used to investigate the transcriptional activity of VvWRKY37 and its binding activities to downstream target genes (Wang F. et al., 2019). The full-length coding sequence of *VvWRKY37* was inserted into the pGADT7 vector to generate the effect or construct, and binding sequence Box 2 (5′-ATTGACTTGACCGTCATCGG-3′) was introduced into the upstream region of the HIS3 mini-promoter to generate the reporter construct. Additionally, yeast one-hybrid (Y1H) screening was performed to search for the putative TFs that were genetically epistatic to *VvWRKY37* (Ouwerkerk and Meijer, 2001). The promoter fragment (1,165 bp) of *VvWRKY37* was sub-cloned and introduced into the upstream region of the minimal HIS3 promoter to generate the bait vector. Total RNA was extracted from the grape vine root, shoot, leaf, and winter bud and used to construct a cDNA expression library (OE Biotech, Shanghai, China). The cDNA library screening was done by transferring the bait vector into the Y187 yeast strain. The putative transformants were identified by their growth on a medium lacking histone, leucine, and tryptophan, and containing 3-aminotriazole (3-at), (Solarbio, Beijing, China). PCR with general primer pairs was run to amplify the individual transformants (Supplementary Table 1). Each PCR product was subjected to DNA sequencing, and a blasting tool from NCBI was used to verify its putative identity.

### Transient Expression Assay

Partially expanded grape vine leaves were used for a transient expression assay *via A. tumefaciens* inoculation as described earlier (Wang F. et al., 2019). Histochemical staining was conducted for a GUS reporter assay after inoculation and *in vitro* co-cultivation for 3–5 days. The dual luciferase was conducted in the living tobacco leaf. The mixed suspension of *A. tumefaciens* harboring effector and reporter constructs was injected into tobacco leaves by using a single-use syringe. The reporter construct was actuated by the promoter of *PtCPY707A2*(2, 000 bp) and *VvWRKY37* (1,165 bp), respectively. The transformed tobacco plants were placed in a dark room for 1 day, followed by 3–5 days under normal growth conditions. Before determining the enzyme activity of luciferase, a tobacco leaf was cut down and sprayed with substrate solution (0.15 mg/ml D-Luciferin Sodium Salt, 0.02% Triton-X 100). After that, the leaf was kept in dark for 5 min at room temperature. Finally, a CCD device was used to visualize fluorescence images of injected leaves.

### Bud Break Assay

Two-year-old poplar plants were prepared for bud break investigation. Before autumn, the transgenic and control (84 K) poplar plants were placed outside under natural environmental conditions until a bud set (at the end of October). The plants were then transferred to an incubator (4°C, dark conditions for 24 h) for 2 months to meet their chilling requirement, and then moved into a greenhouse under a long-day photoperiod (8-h dark/16-h light) at 21°C temperature. The date of bud break (emergence of green tissues at the tip of the bud) was monitored and recorded after every 2 days. The bud, together with surrounding bark, was peeled at two time points: 6 and 12 days. More than 40 buds were collected from six plants for each genotype of poplar plants. The same biological bud pools were used for measuring endogenous ABA and PA content and RT-qPCR analysis of candidate genes. The contents of ABA and PA were determined by liquid chromatography and mass spectrometry at the Institute of Chemistry, Chinese Academy of Sciences (ICCAS, Beijing).

### GUS Staining

GUS activity was carried out by following a previously described protocol (Weigel and Glazebrook, 2002). Plant materials were fully immersed in a 50-ml centrifuge tube containing a staining buffer. The tubes were then kept in a vacuum pump for 10 min to remove air in the plant tissues as much as possible before incubating under dark conditions at 37°C temperature for 12 h. The staining buffer contained 0.5 mg/ml X-gluc (Yeasen, Shanghai, China), a 100-mM phosphate buffer (pH = 7.), 0.01% Triton X-100, 10-mM EDTA-Na_2_, 0.5-mM K_4_Fe (CN)_6,_ and 0.5-mM K_3_Fe (CN)_6_. After incubation, the reaction buffer was replaced with 75% (v/v) ethyl alcohol for 2-3 times to remove chlorophyll. Finally, the fully bleached samples were visualized using a stereoscope (Leica, Wetzlar, Germany).

### Prokaryotic Expression in Recombinant Proteins

The coding sequence of VvWRKY37 without stop codon was cloned in-frame to the pET32a prokaryotic expression vectors (6HIS tag). The homologous recombination method was used to construct VvABF2-HIS and VvABI5-HIS. All gene-specific primer pairs were designed using online software CE design V1.04 (Vazyme, Nanjing, China) (Supplementary Table 1). The recombinant vectors were introduced into E. coli strain TransB/DE3 (TransGen, Beijing, China). Recombinant protein induction was carried out by adding 2-mM isopropyl β-D-1-thiogalactopyranoside (Solarbio, Beijing, China) in the bacterial culture at 37°C for 6 h or at 16°C overnight with constant shaking at 150 rpm. The recombinant protein was purified by ProteinIso^®^ Ni-NTA Resinkit (TransGen, Beijing, China) according to the manufacturer’s protocol. The empty prokaryotic proteins (6HIS) were also induced, purified, and then used as a control in EMSA assays.

### Electrophoretic Mobility Shift Assay

The cDNA fragments, including putative W-boxes and their complementary chains, were synthesized by GENEWIZ (GENEWIZ, Suzhou, China). Each cDNA fragment was labeled with biotin by using the EMSA Probe Biotin Labeling Kit (Beyotime, Shanghai, China). The reverse complements of biotin-labeled oligonucleotides (equally mixed by volume) were annealed by a thermal cycler (Thermo Fisher, Waltham, United States) to form double-stranded DNA using the temperature gradient descent strategy. The non-biotin-labeled wildtype and mutated DNA probes acted as competitors. All primers used in this assay are shown in Supplementary Table 1. A 10 μl of binding reaction mixtures contained: 1-mg purified recombinant protein, a 2-μl 5- × gel shift binding buffer (50% glycerol; 50-mM MgCl_2_; 5-mM EDTA; 10-mM DTT; 500-mM KCl; and 250-mM HEPES; pH, 7.4. A 1-μl (0.05 μM) aliquot of the labeled probe was incubated with the above-mentioned solution at 24°C for 20 min. The binding mixtures were pre-incubated with the unlabeled or mutated probe at 24°C for 20 min and then supplemented with labeled probes for another 20 min for the competition assays. The HIS protein alone was used as the negative control. After incubation, each sample was resolved using 6% non-denaturing acrylamide gel electrophoresis in a 0.5 × TBE buffer (1-mM EDTA-Na_2_, 44.5-mM Tris-base, and 44.5-mM boric acid) at 100 V for 1-2 h. The gel was then transferred to a nylon membrane (GE Healthcare, London, United Kingdom) for chemiluminescent detection (Solarbio, Beijing, China).

### Protein Extraction and Immunoblot Analysis

The crude protein from poplar leaf was extracted using a Plant Protein Extraction Kit (Solarbio, Beijing, China). Total protein concentration was quantified with micro-spectrophotometer ND2000C (Thermo Fisher, Waltham, United States). The tag antibodies, anti-actin, and anti-GFP were purchased from Abmart (Abmart, Shanghai, China) and used according to the manufacturer’s protocol. Immunoblot analysis was carried out by following a previously described protocol (Wang X. et al., 2019).

## Results

### *VvWRKY37* Is Specifically Expressed in Dormant Bud of Grape Vine

**FIGURE 1:**
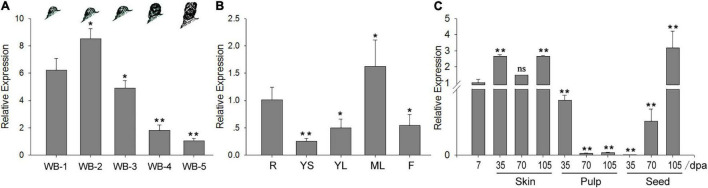
The *VvWRKY37* expression pattern in grape vine. **(A–C)** The relative expression values of VvWRKY37 gene in different grape tissues using qRT-PCR., WB-1: winter buds at the beginning of leaf fall in autumn, WB-2: winter buds in the endodormancy phase, WB-3: winter buds during transition from endodormancy to ecodormancy, WB-4: winter buds at ecodormancy, WB-5: the green tip stage of winter buds, which was defined as bud break. R: root, YS: young stem, YL: young leaf, ML: mature leaf, I: inflorescence (full bloom). *VvActin* is used as the reference gene, and gene expression is normalized to WB-5 **(A)**, root **(B)** or 7 dpa **(C)** expression levels, which are assigned a value of 1. Each datum represents the average of triple biological replicates + SD. Asterisks in panels **(A–C)** indicate statistically significant differences compared with the level of WB-1 **(A)**, root **(B)** or 7 dpa **(C)**: *p < 0.05, **p < 0.01. ns, no significance; dpa, days post-anthesis.

The expression levels of *VvWRKY* TF genes were investigated in different tissues of grapes to characterize their potential involvement in bud dormancy. Interestingly, a *VvWRKY37* exhibited higher expression levels in dormant winter buds of grape vine. We investigated the expression levels of *VvWRKY37* in winter buds at different dormancy phases: paradormancy, endodormancy, ecodormancy, bleeding period, and bud break. The *VvWRKY37* was highly expressed during dormancy establishment in the fall, and then it gradually decreased until bud break in the next spring (Figure 1A). Upon bud break in spring, the expression level of *VvWRKY37* was almost six times lower than that during dormancy initiation in the fall (Figure 1A). Tissue-specific expression analysis showed that *VvWRKY37* was expressed in roots, stems, and leaves (Figure 1B). The RT-qPCR result confirmed that, initially, *VvWRKY37* was highly expressed during the fruit set (7 days post-anthesis), and then it was sharply decreased from 7 dpa to 35 dpa in seeds; finally, it was barely detectable at 35 dpa (Figure 1C). The relative expression levels were markedly raised to 70 times and 50 times in seeds from 35 to 70 dpa (véraison berry) and from 70 to 105 dpa (mature berry), respectively (Figure 1C).

### *VvWRKY37* Responds to Low Temperature, ABA, and GA_3_

**FIGURE 2:**
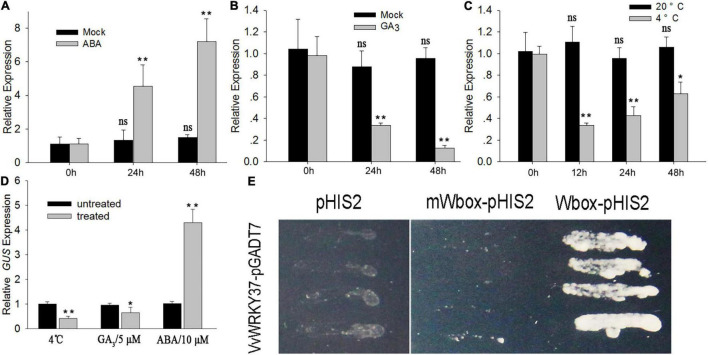
*VvWRKY37* expression responds to exogenous phytohormones and low-temperature treatments. **(A–C)** The relative expression value of *VvWRKY37* in grape buds responding to ABA (10 μM), GA_3_ (5 μM), and low temperature (4°C) treatments. Detached grape vine canes were sampled for different treatments at the beginning of leaf fall. Mock treatment is the control. *VvActin* is used as an internal control, and the default expression value was 1 at 0 h after treatment. **(D)** The transcript abundance of *GUS* gene in the leaf of *pVvWRKY37*:*GUS*. The 14-day-old seedlings were treated with ABA (10 μM), GA_3_ (5 μM), and low temperature (4°C) for 24 h. The 5*^th^* and 6*^th^* leaves were harvested for RNA extraction. *AtGAPDH* is used as an internal control, and the default expression value was 1 for control seedling. Each datum represents the average of triple biological replicates + SD. **(E)** Transcriptional activity of VvWRKY37 on the tandem-repeated BOX2 element, which contains four W-box *cis-*elements. The core motif sequence 5′-TGAC-3′ of W-box was mutated into caAC in m Wbox. The selective medium is nutrition deficiency in histone, leucine, and tryptophan. Asterisks in panels **(A–D)** indicate statistically significant differences compared with 0 h: *p < 0.05, **p < 0.01. ns means no significance.

Plant dormancy is highly associated with environmental and physiological factors, i.e., temperature, photoperiod, and endohormones (Liu and Sherif, 2019). To determine the regulatory role of *VvWRKY37*, the detached canes were treated with exogenous ABA, GA_3_, and low temperature, respectively. Then RT-qPCR was carried out to check the change in expression levels of the *VvWRKY37* in grapevine buds. As expected, the expression level of *VvWRKY37* was significantly higher in ABA-treated cane samples taken at 24 h (h). Additionally, the expression level was 3.5- and 4.8-folds higher than that of the mock treatment at 24 h and 48 h (Figure 2A), respectively. Inversely, GA_3_ application and low-temperature treatment attenuated the mRNA level of *VvWRKY37* in grape vine buds (Figures 2B,C). Under low-temperature treatment, the expression level of *VvWRKY37* decreased from 0 to 12 h and maintained a low level until 48 h (Figure 2C).

To investigate the promoter activity of *VvWRKY37*, the expression of the β-glucuronidase (*GUS*) reporter gene under the control of the *VvWRKY37* promoter was induced in *Arabidopsis via* Agrobacterium-mediated transformation. The positive transformants were detected by selective medium and histochemical GUS staining. The 14-day-old homozygous seedlings were used to evaluate the effects of exogenous ABA and GA_3_, and low temperature on the promoter activity of *VvWRKY37*. The results of *GUS* gene expression (Figure 2D) and histochemical staining (Supplementary Figure 2) in *pVvWRKY37*:*GUS* in *Arabidopsis* showed that exogenous application of ABA induced higher *GUS* expression, while GA_3_ and low temperature repressed *GUS* expression compared with the control plants.

### VvWRKY37 Binds the Canonical W-Box Motif in Yeast

WRKY TFs, one of the largest gene families in plants, play dual transcriptional roles by perceiving and binding the highly conserved *cis*-element W-box TTGACC/T (Rushton et al., 2010). A short DNA sequence, defined as a BOX2 element, was firstly proved to be particularly bound by WRKY TFs (Rushton et al., 1995). To test the transcriptional activity of VvWRKY37, the bait vector was constructed by introducing a tandem-repeated BOX2 element upstream of the reporter gene. Our results showed that yeast transformants harboring the prey vector pGADT7-VvWRKY37 and the bait vector Wbox-pHIS2 grew well on a selective medium (Figure 2E). In contrast, no yeast cell grew on the selection medium when the core motif 5′-TGAC-3′ of the W-box element was mutated (mWbox) into 5′-caAC-3′ (Figure 2E). Thus, VvWRKY37 showed transcriptional activity by binding to the canonical W-box motif. However, it is still unclear whether VvWRKY37 acts as an activator or a repressor in the VvWRKY37-mediated regulatory networks.

### Ectopic Overexpression of *VvWRKY37* Results in Delayed Bud Break in Poplar

**FIGURE 3:**
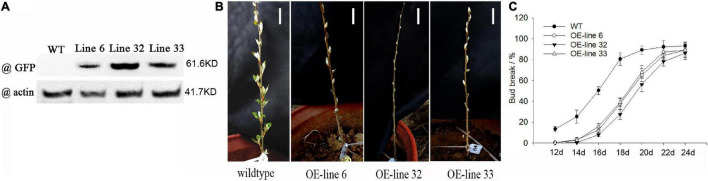
The bud break phenotypes of transgenic plants of VvWRKY37 over-expressing in poplar. **(A)** The level of the VvWRKY37-GFP fusion protein in 35S:VvWRKY37-GFP transgenic poplar lines; an anti-GFP antibody was used in the immunoblot analysis. The anti-actin antibody was used as loading control. The numbers present the protein molecular size. **(B)** Bud-break phenotypes of WT and transgenic poplar plants in spring under environmental conditions. The scale bar is 5 cm. **(C)** The dynamic of bud break in VvWRKY37-overexpressing and control poplars over days after dormancy release. Abscissa is the time (day) after moving the poplars into the greenhouse. The values are the average of means of triple biological replicates of three transgenic lines and for five WT plants. Bars represent SD.

We did heterologous overexpression of *VvWRKY37* in perennial deciduous poplar to explore its biological function in bud dormancy. The leaves of poplar “84K” (*Populusalba* × *Populusgllandulosa*) plants were used to generate stable transgenic plants *via* Agrobacterium-mediated transformation. More than ten independent transgenic poplars were obtained according to the result of PCR amplification (Supplementary Figure 3). Three lines (Line 6, Line 32, and Line 33) were chosen for further investigation, while the wildtype (WT) poplar was used as control. The immunoblot assay verified that the fusion protein VvWRKY37-GFP was substantially overexpressed in three transgenic poplar plants (Figure 3A). The bud break phenotypes were investigated among WT and the transgenic poplar plants after dormancy release under natural conditions. Three transgenic lines exhibited delayed bud break compared with the WT (Figure 3B). The initiation of the endodormancy phase was determined by the bud break dynamics assay. The results showed that heterologous overexpression of *VvWRKY37* impeded the initiation time of bud break, which led to delayed bud break for transgenic poplar plants (Figure 3C). The bud break initiation in transgenic poplar plants was delayed for 4 days compared to WT poplar plants (Figure 3C). Additionally, the delayed efficiency on winter bud break positively related to the gene expression level of *VvWRKY37* in transgenic poplar. The transgenic poplar line 32 with the strongest over-expression of *VvWRKY37* represented the latest bud break during the spring season.

### Abscisic Acid Catabolism Related Gene *PtCYP707A2* Is Repressed in Transgenic Poplar Plants

**FIGURE 4:**
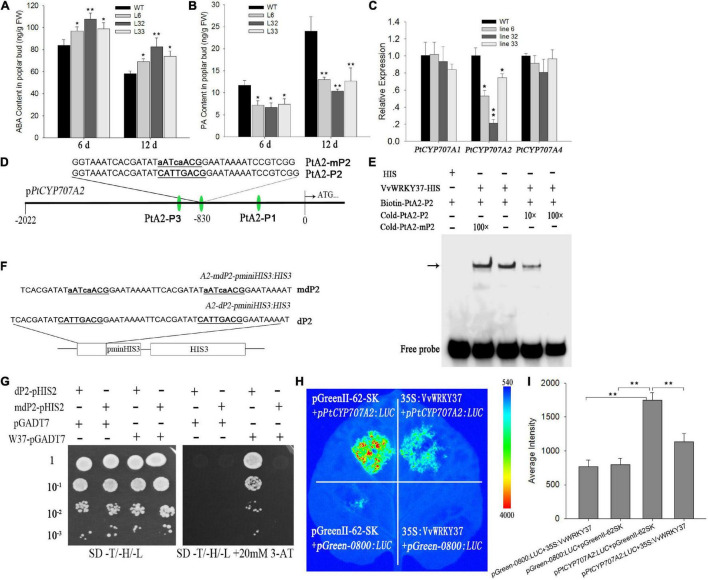
VvWRKY37 affects abscisic acid catabolism. **(A,B)** ABA and PA content in transgenic and WT poplars. Fresh buds were accurately weighed for metabolite quantification. Each datum represents the average of triple biological replicates + SD. **(C)** Expression patterns of *PtCYP707A*s in *35S:VvWRKY37* and WT poplars. *PtUbi* is used as an internal control, and gene expression is normalized to the WT expression level, which is assigned a value of 1. Each datum represents the average of triple biological replicates, and error bars indicate SD. **(D)** Nucleotide sequences of the probes derived from the *PtCYP707A2* promoter are used for VvWRKY37-His recombinant protein-binding assays. The core sequence 5′-CATTGACG-3′ of PtA2-P2 is mutated into 5′-aATcaACG-3′ in PtA2-mP2. **(E)** DNA-binding affinity of VvWRKY37 to W-box fragment PtA2-P2. Competition assays were done with an increasing amount of an unlabeled probe (10-fold and 100-fold). The unlabeled probe mP1 is 100-fold excess of labeled probe P1 in Lane 2 (from left to right). The position of protein-DNA complexes is indicated by an arrow. **(F,G)** VvWRKY37 can bind to the W-box *cis-*element of the *PtCYP707A2* promoter *in vitro* by a yeast one-hybrid assay. The nucleotide fragment A2-dP2 contains two tandem repeats of 5′-CATTGACG-3′, which are both mutated into 5′-aATcaACG-3′ in A2-mdP2. 10^– 1^, 10^– 2^, and 10^– 3^ indicate the yeast concentration diluted with a 0.9% NaCl buffer by 0.1, 0.01, and 0.001. 3-AT stands for 3-Amino-1, 2, 4-triazole. **(H)** Luciferase assays were conducted *via* co-injecting *A. tumifaciens* into *Nicotianabenthamiana* leaves. The activities of luciferase were imaged using a CCD device. **(I)** The average intensity of fluorescence exhibited on the tobacco leaves. The fluorescence values were calculated using the software Image J (1.46 R). Asterisks in panels **(A–C,I)** indicate statistically significant differences as indicated: *p < 0.05, **p < 0.01.

Earlier, we have proved that *VvWRKY37* delayed bud break in transgenic poplar plants. However, there is no reported work available on the signaling pathway modulated by VvWRKY37 in plant dormancy-related regulatory networks. We firstly measured ABA and the catabolite of winter buds in WT and the transgenic poplar plants to better understand their correlation with VvWRKY37. Regardless of bud break status, the ABA levels in buds of transgenic poplar plants were higher than that of WT poplar plants (Figure 4A). In contrast, the level of the ABA catabolite phaseic acid (PA) was significantly decreased in transgenic plants compared with WT poplar plants (Figure 4B). We analyzed transcript levels for central components involved in ABA biosynthesis and catabolism to detect the molecular mechanism behind increased levels of ABA in the transgenic poplar plants. Our results showed that the expression level of *PtCYP707A2* was significantly downregulated in the transgenic poplar plants (Figure 4C). The expression level of *PtCYP707A2* intransgenic line 32 was 5-fold lower than that of the WT poplar plants. However, no change in the expression level was observed for two other paralog genes *PtCYP707A1* and *PtCYP707A4* (Figure 4C). Additionally, no noticeable difference was observed in the expression level of ABA biosynthesis genes, *PtNCED1*, *PtNCED3*, *PtNCED5*, and *PtNCED6* between the WT and transgenic poplars plants (Supplementary Figure 4).

The above-mentioned findings suggested that *VvWRKY37* possibly enhanced ABA accumulation *via* transcriptional repression of ABA catabolic genes. Firstly, we explored available evidence to verify whether VvWRKY37 directly regulated *PtCYP707A2*. The results of the transcriptional activity assay showed that VvWRKY37 could bind to the W-box *cis*-element (Figure 2E). The *cis*-regulatory element analysis in the upstream promoter region of the *PtCYP707A2* gene resulted in identification of three candidate W-box elements. Each of the W-box elements, together with the flanking sequence, was designed as a DNA probe and named as PtA2-P1, PtA2-P2, and PtA2-P3, respectively (Figure 4D and Supplementary Figure 5A). The recombinant protein VvWRKY37-HIS was prepared by using a prokaryotic expression method. The electrophoretic mobility shift assay (EMSA) results indicated that VvWRKY37 specifically interacted with the DNA probe PtA2-P2, and the competitive assay further confirmed the protein-DNA interaction *in vitro* (Figure 4E and Supplementary Figure 5B). The core motif of the W-box was irreplaceable for the proper interplay between VvWRKY37 and DNA probe PtA2-P2 (Figure 4E). The EMSA results were cross-checked by using the Y1H assay (Figure 4F). The yeast cells harboring VvWRKY37 and wildtype W-box motifs grew well on the selective medium, while the negative control could not grow (Figure 4G). The VvWRKY37 failed to activate the reporter gene in yeast cells when the W-box motif 5′-CATTGACG-3′ was mutated into 5′-aATcaACG-3′ (Figure 4G). The transient expression assay showed that heterologous overexpression of VvWRKY37 in tobacco leaves decreased luciferase gene expression initiated by the promoter of *PtCYP707A2* (Figures 4H,I). Co-transforming *35S*:*VvWRKY37* with *pPtCYP707A2*:*LUC* significantly attenuated luciferase activity compared with the empty vector (Figures 4H,I). The VvWRKY37 did not depress the expression of the luciferase gene initiated by the mutant promoter of *PtCYP707A2* on the deletion of W-box from it, indicating that the W-box motif was indispensable for VvWRKY37 modulating the promoter activity of *PtCYP707A2* (Supplementary Figure 6). Therefore, from the above analysis, we inferred that VvWRKY37 can directly bind to the promoter region of *PtCYP707A2* and transcriptionally repressed the promoter activity.

### VvWRKY37 Binds to the Promoter of *VvCYP707A4.2* and Suppresses Its Expression in Grape Vine

**FIGURE 5:**
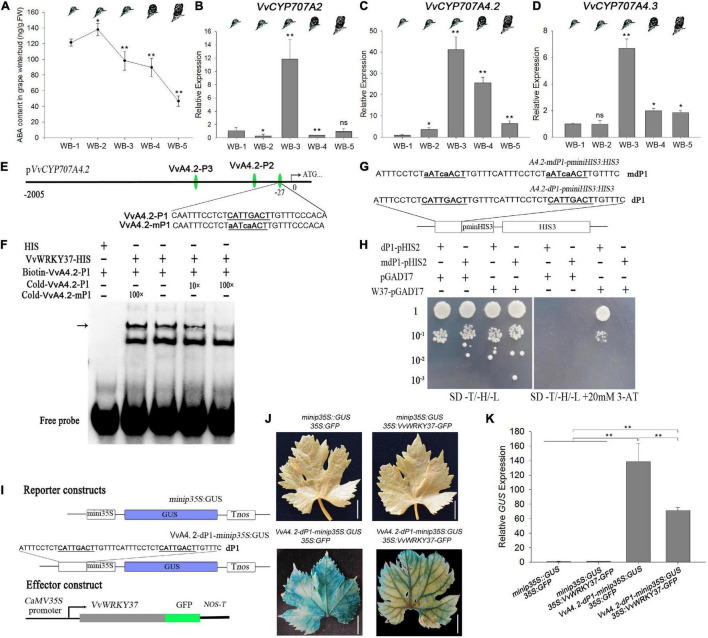
VvWRKY37 modulates the expression of *VvCYP7074.2* in grape vine. **(A)** ABA content in grape vine winter buds at different dormancy phases. Data are means of five biological replicates ± SD. **(B–D)** *VvCYP707A2*, *4.2*, and *4.3* expressions in grape winter buds at different dormancy phases. *VvActin* serves as the reference gene, and the mRNA abundance is normalized to the WB-1-stage expression level assigned a value of 1. Each datum represents the average of triple biological replicates + SD. **(E)** Nucleotide sequences of the DNA probes derived from *VvCYP707A4.2* promoter. The W-box *cis-*element 5′-CATTGACT-3′ of VvA4.2-P1 is mutated into 5′-aATcaACT-3′ in VvA4.2-mP1. **(F)** The EMSA assay was employed to test DNA-binding affinity of VvWRKY37 to the W-box fragments VvA4.2-P1. Competition assays were performed just as described in Figure 4E. The position of protein-DNA complexes is indicated by an arrow. **(G,H)** VvWRKY37 can bind to the W-box *cis-*element of the *VvCYP707A4.2* promoter *in vitro* by a yeast one-hybrid assay. The nucleotide fragment A4.2-dP1 contains two tandem repeats of 5′-CATTGACT-3′, which are both mutated into 5′-aATcaACT-3′ in A4.2-mdP1. 10^–1^, 10^–2^, and 10^–3^ indicate the yeast concentration diluted with a 0.9% NaCl buffer by 0.1, 0.01, and 0.001. 3-AT stands for 3-Amino-1, 2, 4-triazole. **(I)** Schematic diagrams of the reporter and effector constructs used in the transient co-transformation of grape vine leaves *via A. tumifaciens*. The nucleotide fragment A4.2–dP1 contains two tandem repeats of W-box *cis-*element 5′-CATTGACT-3′, which are both mutated into 5′-aATcaACT-3′ in A4.2–mdP1. **(J)** The images of GUS-stained grape vine leaves after injecting *A. tumifaciens*. All white scale bars indicate 1 cm. **(K)** The transcript level of *GUS* gene in transformed grape vine leaves. Asterisks in panels **(A–D,K)** indicate statistically significant differences as indicated: *p < 0.05; **p < 0.01. ns means no significance compared with WB-1.

The expression of *VvWRKY37* was induced in response to the exogenous application of ABA (Figure 2A), which implied the potential relationship between *VvWRKY37* and ABA levels in the grape vine. The ABA contents of grape vine buds were measured from paradormancy to bud break. The data presented in Figure 5A suggested that the highest ABA level was found at the time of bud dormancy establishment in the winter. The ABA content was reduced in grape vine winter buds from the endodormancy phase to bud break. It was likely that the expression level of *VvWRKY37* was directly proportional to the ABA level during bud dormancy periods (Figures 1A, 5A). The RT–qPCR results illustrated that relative expressions of *VvCYP707A2*, *4.2*, and *4.3* exhibited dramatic changes in grape vine winter buds from dormancy establishment to bud break (Figures 5B–D). The winter bud presented a relatively low relative expression level of *VvCYP707A2*, *4.2*, and *4.3* from dormancy establishment (WB-1) to the endodormancy phase (WB-2), while it sharply increased after the endodormancy phase (WB-3) (Figures 5B–D). From the end of the endodormancy phase to bud break, the relative expression of *VvCYP707A2*, *4.2*, and *4.3* was downregulated to an extremely low level (Figures 5B–D). The relative expression levels of *VvCYP707A2*, *4.2*, and *4.3* reached to a peak at the end of the endodormancy phase.

We carried out some additional assays to verify the molecular relationship between VvWRKY37 and *VvCYP707A4.2*. The binding affinity between VvWRKY37 and W-box *cis*-element adjacent to the transcription start site of *VvCYP707A4.2* was confirmed by both EMSA and Y1H assays (Figures 5E–H). The EMSA assay results showed that VvWRKY37 could not bind to the other two W-box motifs (Supplementary Figures 7A,B). Interestingly, we found that the core motif of W-box *cis*-element (5′-CATTGACG/T-3′) bound by VvWRKY37 was similar to the core motif (5′-GATTGACT-3′) in tandem-repeated Box 2, which was tested earlier in the transcription activity assay (Figure 2E). We failed to find a similar motif in the promoter regions of *PtCYP707A1*, *PtCYP707A4*, *VvCYP707A2*, and *VvCYP707A4.3* (Supplementary Table 2). Preliminary evidence supported our argument that 5′-G/CATTGACG/T-3′ might be the conserved binding preference for VvWRKY37. A transient expression assay was conducted to verify the inhibitory effect of VvWRKY37 on the W-box motifs in the promoter region of *VvCYP707A4.2* (Figure 5I). The reporter construct contained a tandemly repeated W-box motif of *VvCYP707A4.2*, which was inserted in the front of the mini CaMV35S promoter. The recombinant plasmid *35S*:*VvWRKY37-GFP* and the reporter construct Vv4.2-dP1-*minip35S*:*GUS* significantly attenuated the accumulation of GUS protein in grape vine leaves compared with the empty vector *35S*:*GFP* (Figure 5J). The RT-qPCR results also confirmed that VvWRKY37 noticeably depressed the expression of reporter gene *GUS* (Figure 5K). The expression analysis results for transformed grape vine leaves showed that overexpression of *VvWRKY37* significantly downregulated the expression levels of *VvCYP707A4.2*, while the expression level of *VvCYP707A4.2* was barely altered in grape vine leaves transformed by the empty vector *35S*:*GFP* (Supplementary Figures 8A,B).

### VvWRKY37 Is Transcriptionally Modulated by the Upstream Regulators of the ABA Signaling Pathway

ABA plays a dominant role in plant dormancy regulatory network signaling cascades through ABA-related regulators. Our results provided substantial evidence to prove that the higher levels of ABA evoked the expression of *VvWRKY37*, while VvWRKY37 inversely affected ABA homeostasis through a feedback loop. However, the mechanism by which the ABA alters the expression level of *VvWRKY37* is still unknown. There could be special factors that regulated ABA homeostasis and transduced downstream signals to the TF VvWRKY37. Firstly, we investigated the *cis*-elements in the promoter region of *VvWRKY37* using an online database (Lescot et al., 2002). Several ABRE (ABA-responsive elements) motifs were identified, implying that *VvWRKY37* was potentially regulated by ABFs (ABRE-binding factors). The ABFs, A subfamily of bZIP TFs induced by exogenous application of ABA, were positively involved in the ABA signal transduction cascade (Kim et al., 2004). We performed the Y1H library screening assay to determine whether VvWRKY37 was modulated by bZIP protein or other TFs. A DNA fragment of the *VvWRKY37* promoter was cloned in-frame to the bait vector, which was then co-transformed with grape vine mRNA library vectors. Finally, we identified a group of ripening-related proteins and an arm repeat protein (Supplementary Table 3). The arm repeat protein has been previously reported to interact with ABF2 and modulate the transcriptional activity of ABF2 (Kim et al., 2004). Thus, VvABF2 and its homeotic gene VvABI5 were considered intermediates that linked the ABA signal and *VvWRKY37* mRNA abundance in the dormancy regulatory network. A series of analyses were carried out to find enough evidence in support of the upstream regulator of *VvWRKY37*.

**FIGURE 6:**
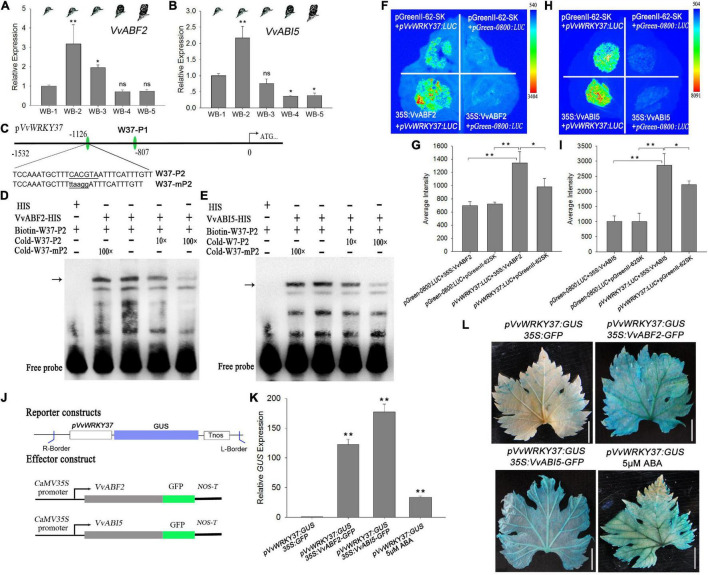
VvWRKY37 is implicated in ABA cascade response *via* upstream regulator VvABF2 and VvABI5. **(A,B)** The mRNA levels of *VvABF2* and *VvABI5* in grape winter buds at different dormancy phases. *VvActin* is used as an internal control, and gene expression is normalized to WB-1-stage expression levels, which were both assigned a value of 1. Each datum represents the average of triple biological replicates + SD. **(C)** Nucleotide sequences of the DNA probes W37-P2 derived from *VvWRKY37* promoter were used for EMSA assays. W37-P2 contains a putative ABRE *cis-*element 5′-CACGTA-3′, which is mutated into 5′-ttaagg-3′ in W37-mP2. **(D,E)** Electrophoretic mobility shift assays for detecting the binding affinity of VvABF2-His **(D)** and VvABI5-His **(E)** fusion proteins. Competition assays were performed just as described in Figure 4E. The position of protein-DNA complexes is indicated by a black arrow. **(F,H)** Luciferase assays were conducted in *Nicotianabenthamiana* leaves *via A. tumifaciens* as described in Figure 4H. The full length promoter of *VvWRKY37* was used in the assays. **(G,I)** The average intensity of fluorescence exhibited on the tobacco leaves. All data are shown as the average of triple biological replicates + SD. **(J)** Schematic diagrams of the reporter and effector constructs for transient expression in grape vine leaves. **(K)** The relative expression of GUS gene in grape vine leaves; *VvActin* is used as an internal control. Each datum represents the average of triple biological replicates + SD. **(L)** Histochemical staining for detecting GUS protein expression in grape vine leaves. Scale bars = 1 cm. Asterisks in panels **(A,B,G,I,K)** indicate statistically significant differences as indicated: *p < 0.05, **p < 0.01. ns means no significance compared with WB-1.

The expression level of *VvABF2* and *VvABI5* reached to peak at the bud dormancy establishment stage and then continuously declined to a low level until the bleeding period (Figures 6A,B), which was almost in parallel to the change of *VvWRKY37*expression levels (Figure 1C). To conduct the EMSA assay, ABRE elements in the promoter region of *VvWRKY37* was designed as DNA probe W37-P1 and W37-P2 (Figure 6C). Both VvABF2 and VvABI5 genes were capable of binding with biotin-labeled DNA fragment W37-P2 but failed to bind with W37-P1 (Figures 6D,E and Supplementary Figure 9). The cold mutant DNA probe Cold-mP2 could not competitively subdue the shifted protein-DNA complex, implying a distinctive binding activity of VvABF2 and VvABI5. Generally, VvABF2 and VvABI5 were considered inducible by the ABA signal and transcriptionally provoked the expression of their downstream target *VvWRKY37*. The transient expression assay confirmed that heterologous overexpression of VvABF2 or VvABI5 in tobacco leaves elicited an enhanced expression of the luciferase reporter gene initiated by the promoter of *VvWRKY37* (Figures 6F,H). Co-transformation of *35S*:*VvABF2* or *35S*:*VvABI5*, together with *pVvWRKY37*:*LUC*, markedly increased luciferase activity than that of the empty effector vector in tobacco leaves (Figures 6G,I). Both VvABF2 and VvABI5 did not show an inhibitory effect on the luciferase activity when replacing the ABRE motif of the *VvWRKY37* promoter (Supplementary Figures 10A,B). The result indicated that the ABRE motif was indispensable for proper interplay between VvABI5 or VvABF2 and *VvWRKY37*.

We also tested the transcriptional activity of VvABF2 and VvABI5 in grape vine leaves *via* the *Agrobacterium*-mediated transformation method. The schematic diagrams of the reporter and effector constructs are shown in Figure 6J. The overexpression of VvABF2 or VvABI5 stimulated a strong expression of the *GUS* reporter gene initiated by the *VvWRKY37* promoter (Figures 6K,L). It seemed that the ABA-inducible TFs, VvABF2, and VvABI5 transcriptionally activated the promoter of *VvWRKY37* through direct protein-DNA interactions. The grape vine leaves inoculated with *pVvWRKY37*:*GUS* accumulated much more GUS protein than under ABA application (Figures 6K,L). The RT-qPCR assay was used to measure the changes in transformed grape vine leaves. Overexpression of VvABF2 or VvABI5 increased the expression level of *VvWRKY37* in grape vine leaves (Supplementary Figures 11A–C). Likewise, the expression level of *VvWRKY37* in transformed leaves was significantly induced by ABA application.

## Discussion

**FIGURE 7:**
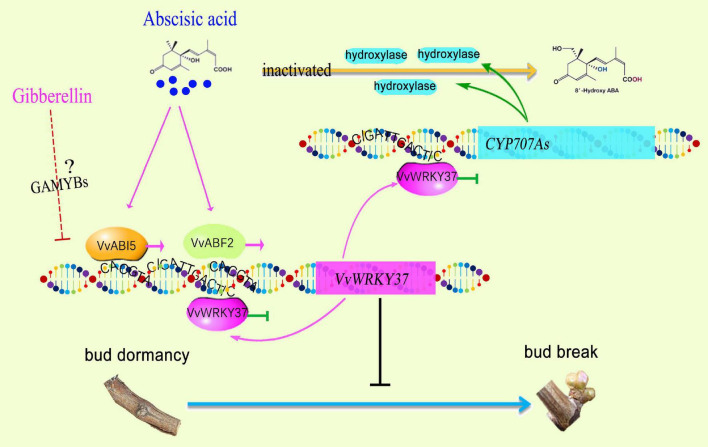
A proposed model for the role of VvWRKY37 in ABA-mediated dormancy regulatory cascades.

Plant WRKY TFs, one of the largest TF families, are increasingly attracting researchers to explore their novel functions, including phytohormone-related biological processes (Rushton et al., 2012; Ding et al., 2014; Liu et al., 2015; Chen et al., 2016; Huang et al., 2016; Zhou et al., 2020). Plant bud dormancy induction, duration, and release were directly associated with environmental conditions, photoperiod, temperature, and endogenous phytohormones (Liu and Sherif, 2019). Genome-wide expression analysis and comparative RNA-Seq analysis have indicated the regulatory role of several *WRKY* genes underlying bud dormancy in perennial plants (Chen et al., 2016; Khalil-Ur-Rehman et al., 2017). However, there is no reported knowledge available on intricate molecular regulatory mechanisms for WRKY TF in bud break. Here, we reported *VvWRKY37*, which was highly expressed in the dormant winter bud of the grape vine. Ectopic overexpression of *VvWRKY37* delayed bud break in transgenic poplar plants. A series of experiments provided a new perspective in which VvWRKY37 modulated bud break through ABA-mediated signaling cascades. Functional validation of VvWRKY37 built a bridge between phytohormone ABA and downstream gene function in plant dormancy regulatory cascade (Figure 7).

Previously, 20 *WRKY* genes related to bud dormancy have been identified by transcriptomic approach from different dormancy phases, summer buds, and winter buds during paradormancy and endodormancy (Khalil-Ur-Rehman et al., 2017). Among these differentially expressed *VvWRKY*s, the expression level of *VvWRKY37* (LOC100267688) decreased at the onset of the endodormancy phase in grape vine buds (Khalil-Ur-Rehman et al., 2017). Coincidently, the expression level of *VvWRKY37* declined continuously from endodormancy to bud break in grape vine winter buds (Figure 1A). The dominant roles of ABA in modulating plant dormancy have been reported in numerous former pieces of literature (Tylewicz et al., 2018; Liu and Sherif, 2019). For an instance, exogenous application of ABA has inhibitory effects on the dormancy release of grape vine buds (Zheng et al., 2015. Typically, the ABA content of grape vine buds increases prior to the endodormancy phase at the onset of autumn (Rubio et al., 2019). Similarly, higher ABA levels are vitally important in avoiding viviparous seeds before release from the mother plant (Kushiro et al., 2004). Our results showed that change in the ABA content was directly proportional to expression levels of *VvWRKY37* in grape vine buds (Figures 1A, 5A), implying an underlying regulatory mechanism. Additionally, we have provided evidence for induction of *VvWRKY37* gene under exogenous application of ABA, and its regulatory role in inversely modulated ABA homeostasis by repressing the catabolism of rate-limiting enzymatic genes *CYP707A*. ABA metabolism is an essential pathway in the ABA-mediated regulation of plant dormancy (Zheng et al., 2015). Overexpression of *VvA8H-CYP707A4* in grape vine reduced the ABA level and enhanced bud dormancy release (Zheng et al., 2018a). Previously, it has been reported that the *NCED* and *ABA8*′*OH* catalytic genes that determine ABA homeostasis are transcriptionally regulated by WRKY TFs (Liu et al., 2015; Luo et al., 2017). In the current study, the expression patterns of *WRKY37* and *CYP707A*s were not highly correlated in grape vine winter buds (Figures 1A, 5B–D). At least, WRKY37*-*mediated transcriptional repression of *CYP707A*s was not found from the bleeding period to bud break. All their transcript abundances were relatively low during this stage. The actual regulatory mechanism might be more complex than our current findings. However, we provided direct evidence to prove that *VvWRKY37* enhanced ABA accumulation *via* repressing the ABA catabolic pathway, but not regulating ABA biosynthesis.

WRKY TFs were characterized for recognizing and binding the *cis*-element 5′-TTGACT/C-3′ (W-box) with an invariant core motif 5′-TGAC-3′ of downstream targets (Rushton et al., 2010). However, the adjacent DNA sequences outside the core motif are sometimes conclusive for the specificity of WRKY-binding affinity (Ciolkowski et al., 2008). The flanking nucleotide is crucial for individual WRKY TF to determine which W-box it should specifically bind in the complex context (Ciolkowski et al., 2008). The binding affinity of the fused protein VvWRKY37-HIS to various DNA probes with different flanking sequences was widely investigated *via* EMSA assays. The VvWRKY37 exhibited a binding affinity to the 5′-G/CATTGACT/C/G-3′*cis*-element (Supplementary Figure 12). Notably, this binding behavior occurred not only in grape vine but also in heterologous plant species, i.e., poplar. It has been speculated that the preferential binding affinity of VvWRKY37 could have originated from lower plant species. Furthermore, dissecting the binding preference of VvWRKY37 could identify the downstream targets involved in VvWRKY37-like TFs-mediated regulatory pathways.

The phytohormone GA promotes seed germination (Née et al., 2017), but its effect on bud break is not absolutely explicit (Zheng et al., 2018b). Although we did not pay much attention to the roles of VvWRKY37 in GA-mediated or chilling-related dormancy regulation, our results imply that VvWRKY37 might be involved in the above-mentioned regulatory cascades. The exogenous application of GA and low-temperature exposure reduced the expression level of *VvWRKY37* in the grape vine (Figures 2B,C). The GA and low temperature acted as bud dormancy release stimulators by directly inhibiting the expression of *VvWRKY37*, or by antagonizing ABA biosynthesis or/and signaling (Seo et al., 2006). However, it was inconsistent that the GA_1_ level in grape vine buds declined from dormancy induction to maintenance (Zheng et al., 2018b), while *VvWRKY37* was downregulated. Perhaps, ABA played a predominant role in modulating VvWRKY37 expression during dormancy initiation and duration, which dissembled the inhibitory effect induced by GA. During bud break, the content of ABA was stable at a low level, while increasing GA-depressed *VvWRKY37* expression. Thereby, the expression level of *VvWRKY37* was downregulated with decreasing ABA levels in grape vine buds before bud break. A previous study has reported that ABA and GA had an antagonistic effect on the regulation of several biological processes (Gazzarrini et al., 2004). Thus, the interplay between the hormones determines their effectiveness.

ABA modulated the expression of *VvWRKY37*, possibly *via* the ABA-stimulated expression of ABI5 and ABF2 (Figure 6). Notably, ABA exhibited a traditional signaling transduction cascade response from signaling molecules to downstream functional genes. Despite the monodirectional top-to-bottom signaling cascade, several secondary regulatory strategies, i.e., the feedback loop, synergistically made the signaling pathway work optimally, especially for hormone cascade responses (Fukazawa et al., 2017; Wang X. et al., 2019). Here, we found that high levels of ABA-induced *VvWRKY37* expression and, in turn, VvWRKY37 feedback enhanced ABA accumulation *via* transcriptional repression of the ABA catabolic gene. It was a positive feedback loop that maintained the triggered role of ABA in *VvWRKY37* expression. However, no unique W-box *cis*-element bound by VvWRKY37 was found in the promoters of ABI5 and ABF2, indicating a monodirectional relationship between VvABI5 or VvABF2 and *VvWRKY37*. VvABI5 and VvABF2 might have redundant functions in regulating *VvWRKY37* because their binding sites on the promoter of *VvWRKY37* were identical, and both of them activated the *VvWRKY37* promoter.

As an effective regulatory pathway, a homeostasis mechanism must be in place to avoid the inhibitory effect of VvWRKY37 on target genes. Previously, WRKY TF has been shown to be transcriptionally self-regulated *via* direct protein-DNA interaction in *Arabidopsis* (Mao et al., 2011). Two adjacent W-box *cis*-elements, 5′-CATTGACC-3′ and 5′-CATTGACT-3′, were identified in the promoter of *VvWRKY37*. As expected, VvWRKY37 showed high-binding affinity to both W-box motifs, which triggered a suppression effect on the reporter gene (Supplementary Figure 13). These results suggest that the expression level of *VvWRKY37* could be modulated *via* a negative feedback loop. These fine-tuned feedback regulations evoked by VvWRKY37 increased our understanding of the complex dormancy-related signal transduction cascades. Overall, we comprehensively dissected the transcriptional roles of VvWRKY37 in the ABA-mediated bud dormancy signal transduction cascades, which unravel novel insights into the molecular regulatory mechanisms underlying different plant dormancy phases.

## Data Availability Statement

The original contributions presented in this study are included in the article/Supplementary Material, further inquiries can be directed to the corresponding author.

## Author Contributions

F-PW designed the study, conducted the experiments, analysis and interpretation of the data, drafted or revised the article. P-PZ conducted the experiments, analysis, and interpretation of the data. LZ revised the article. HZ conceptualized and designed the study. MA revised the article. Y-PD conceived and designed the study and drafted or revised the article. All authors contributed to the article and approved the submitted version.

## Conflict of Interest

The authors declare that the research was conducted in the absence of any commercial or financial relationships that could be construed as a potential conflict of interest.

## Publisher’s Note

All claims expressed in this article are solely those of the authors and do not necessarily represent those of their affiliated organizations, or those of the publisher, the editors and the reviewers. Any product that may be evaluated in this article, or claim that may be made by its manufacturer, is not guaranteed or endorsed by the publisher.
