# Supplementary material for: The VvWRKY37 Regulates Bud Break in Grape Vine Through ABA-Mediated Signaling Pathways
Source: Front Plant Sci. 2022 Jun 16;13:929892. doi: 10.3389/fpls.2022.929892 (PMC9245421; doi:10.3389/fpls.2022.929892)
Supplement: Supplementary file 1 [file Data_Sheet_1.docx]

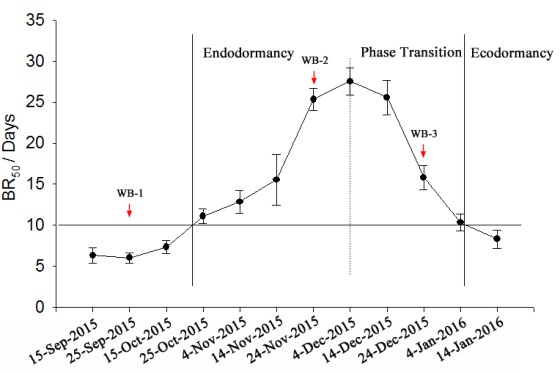


Figure S1 The bud dormancy phases under environmental conditions. On each collection date, a total of 60 single-note cuttings were prepared from 15-Sep-2015 to 14-Jan-2016 on a 10-days interval time. Three groups of 20 cuttings were mounted on a polypropylene sheet and floated in sterile water in a plastic box (length, 40 cm; width, 20cm; height, 15cm). The box was placed in a growth chamber set at 23 ± 2°C with a 16 h photoperiod. The water was replaced every 5 days. Bud break was assayed every 3 days for a period of 30 days. The date was recorded when 50 percentage of buds broke in one group. The red arrows indicate the sampling time for testing transcript abundance of interested genes. Values are averages of three triple biological replicates, and error bars indicate SD.

,


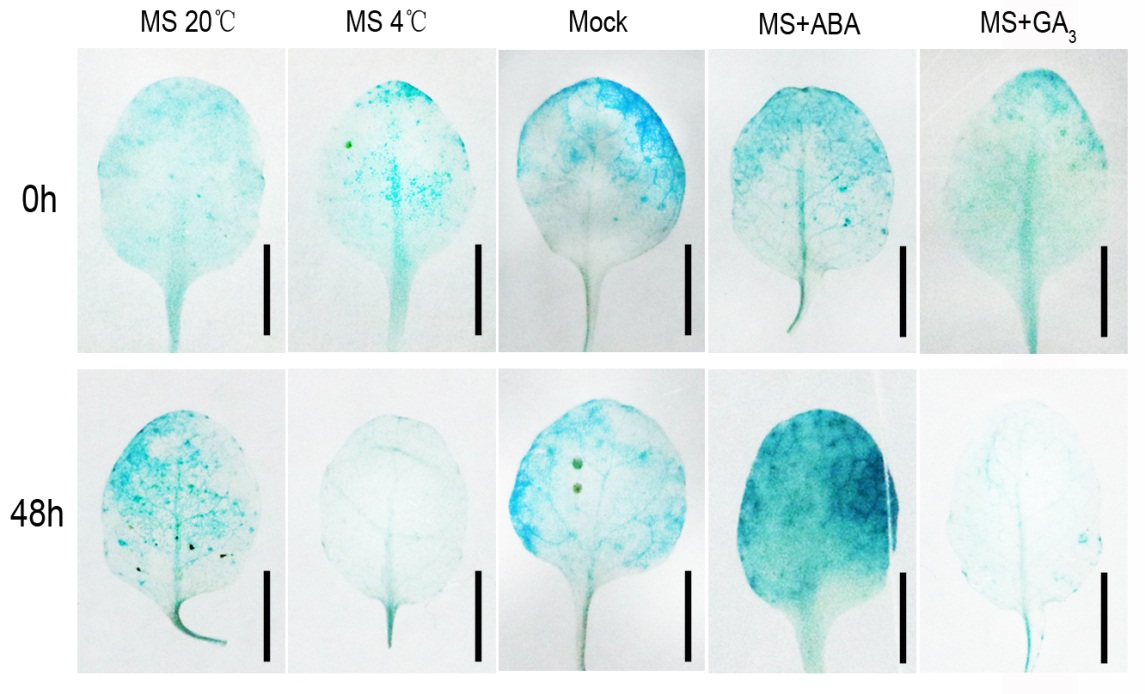


Figure S2 GUS expression in the leaf of transgenic *Arabidopsis* *pVvWRKY37*: GUS after treatments. Transgenic *Arabidopsis* seeds were grown for 5 days on MS medium and transferred to plastic pots filled with a 50% peat and 50% perlite mixture. 10 days later, health seedlings were sprayed with ABA solution (10 μM, 0.06% athanol, 0.02% Triton X-100) or GA3 solution (5μM, 0.06% athanol, 0.02% Triton X-100). The control seedling was sprayed with mock solution (0.06% athanol, 0.02% Triton X-100). Several seedlings were transferred to an incubator for low temperature (4 °C) treatment while 20 °C treatment served as control. 24 hours after treatment, the whole seedlings were harvested for GUS staining. The 5^th^ or 6^th^ leaf was cut off and photoed. All black scale bars are 1 mm.


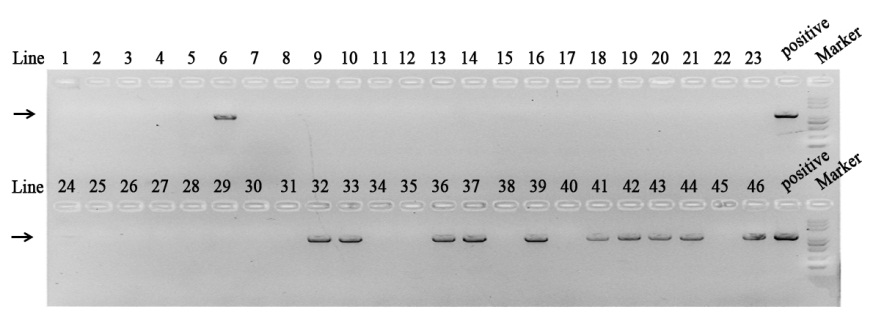
 Figure S3 Genotype analysis of T0 t ransgenic poplars using PCR. The total DNA was extracted from the leaf of T0 transgenic poplars (46 independent transgenic lines) using CTAB method. Each PCR reaction volume contains 1 μl DNA solution. The forward and reverse primer used for genotype is 5′-CCACTATCCTTCGCAAGACCCT-3′ and 5′-CTTCACACCCTCCTCCCTGC-3′, respectively. The plasmid 35S:VvWRKY37-GFP was added as positive template. The PCR amplification procedure was as follows: initial [denaturation](http://www.sciencedirect.com/topics/biochemistry-genetics-and-molecular-biology/denaturation-biochemistry) at 94 °C for 5 m, followed by 32 cycles at 94 °C for 5 s, 50 °C for 30 s, and 72 °C for 30 s. The black arrow indicated the target band in the gel. The DNA marker is 5000bp, 3000bp, 2000bp, 1000bp, 750bp, 500bp, 300bp and 100bp.


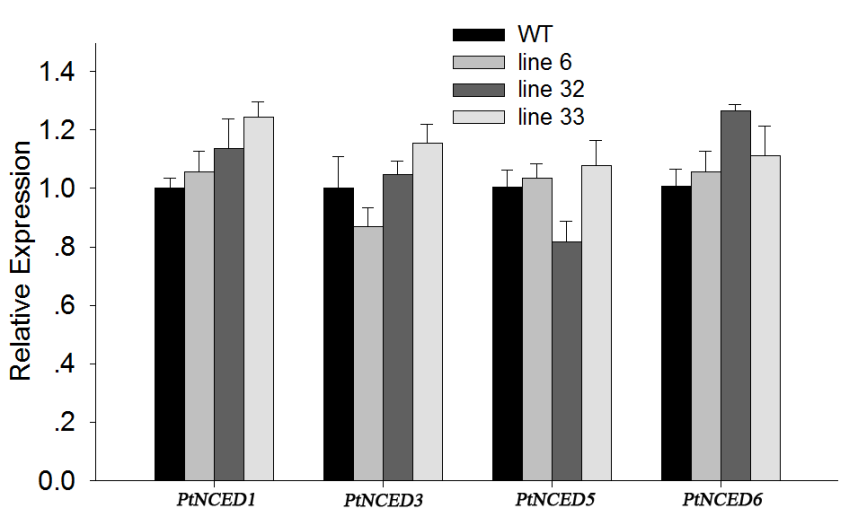


Figure S4 The transcript abundance of *PtNCED*s in transgenic poplars. *PtUbi* is used as an internal control and gene expression is normalized to the WT expression level, which is assigned a value of 1. Each data represents the average of triple biological replicates, and error bars indicate SD.


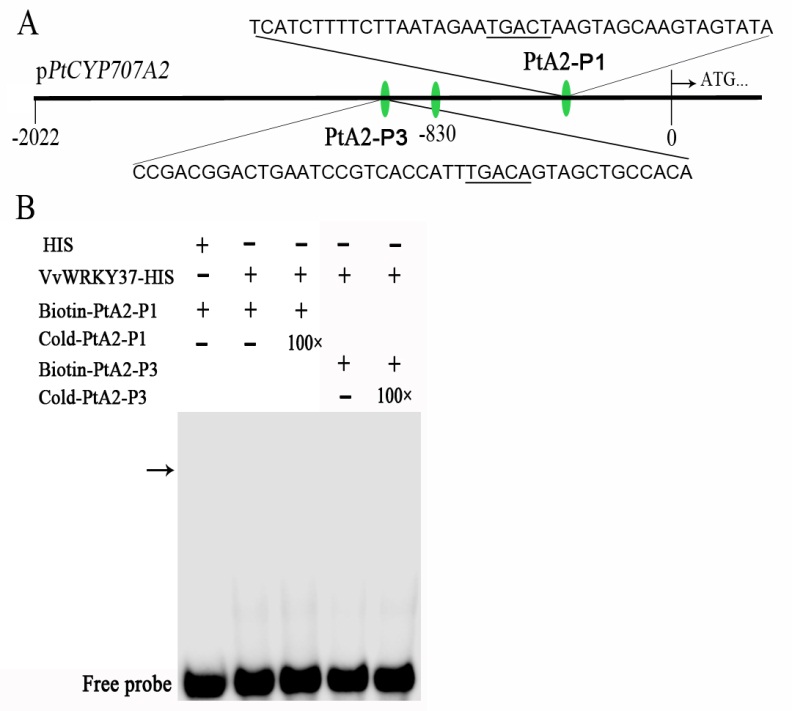


Figure S5 DNA binding affinity of VvWRKY37 to DNA probes of *PtCYP707A2* *in vitro* using EMSA assay. Unlabeled probes were used as the competitors with a concentration of 100-fold of labeled probes. The probes presented here could not be bound by recombinant proteins VvWRKY37-HIS. The putative position of protein-DNA complexes is indicated by an arrow.


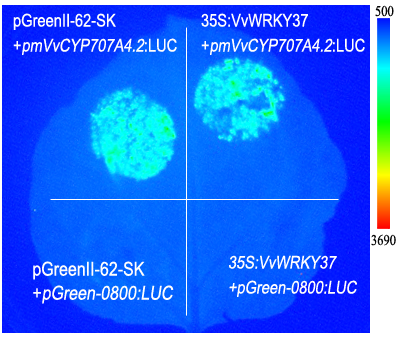


Figure S6 The activity of mutant promoter of *VvCYP707A4.2* in tobacco leaf. The promoter of *VvCYP707A4.2* was mutated before inserting into the reporter construct, which made failed interplay between VvWRKY37 and the promoter of *VvCYP707A4.2* in tobacco leaf. The activities of luciferase were imaged using a CCD device.


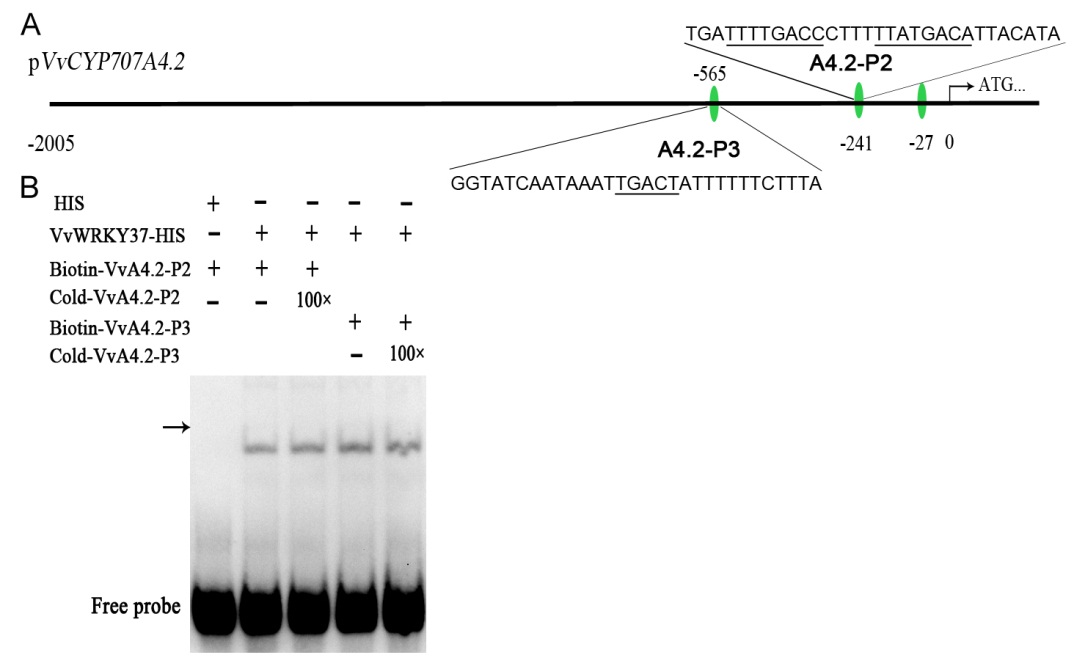


Figure S7 DNA binding affinity of VvWRKY37 to DNA probes of *VvCYP707A4.2* *in vitro* using EMSA assay. Unlabeled probes were used as the competitors with a concentration of 100-fold of labeled probes. The probes presented here could not be bound by recombinant proteins VvWRKY37-HIS. The putative position of protein-DNA complexes is indicated by an arrow.


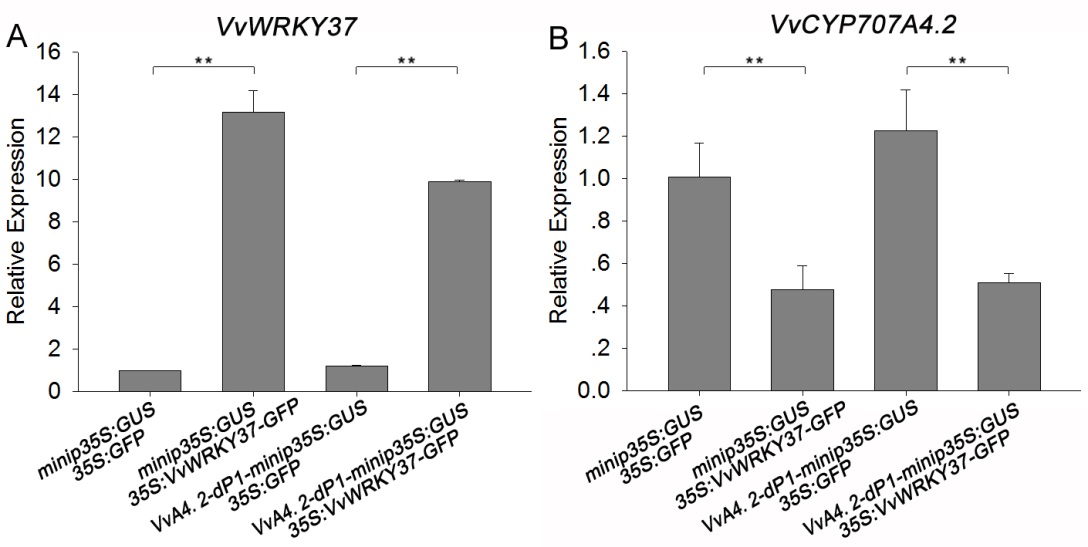


Figure S8 The mRNA levels of *VvWRKY37* and *VvCYP70A4.2* in transiently transformed grapevine leaves. *VvActin* serves as the reference gene. Asterisks indicate statistically significant differences as indicated: **, P < 0.01.


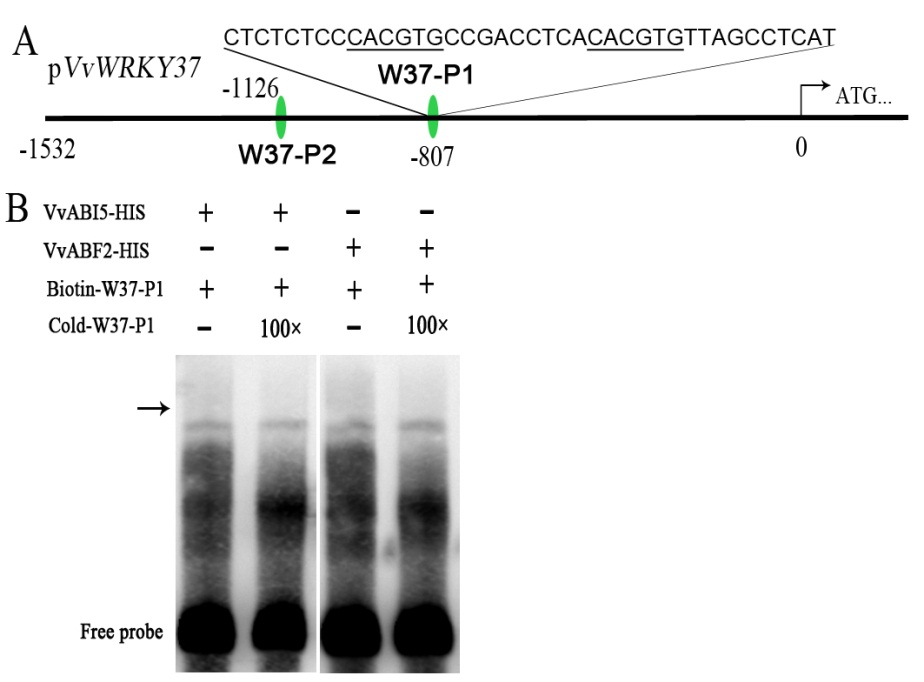


Figure S9 DNA binding affinity of fusion proteins VvABI5-HIS and VvABF2-HIS to indicated DNA probe *in vitro* using EMSA assay. Unlabeled probe (Cold-W37-P1) was used as the competitor with a concentration of 100-fold of biotin labeled probe. The putative position of protein-DNA complexes is indicated by the black arrow.


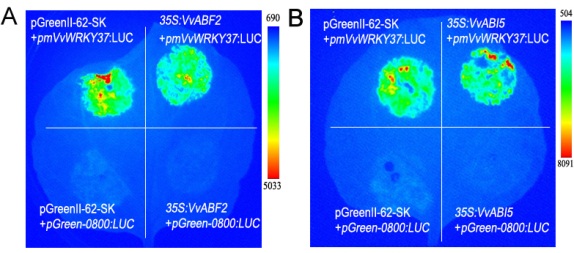


Figure S10 The activity of mutant promoter of *VvWRKY37* in tobacco leaf. The promoter of *VvWRKY37* was mutated before inserting into the reporter construct, the ABRE *cis*-element 5′-CACGTA-3′ was replaced with 5′-ttaagg-3′. This mutant made failed interplay between VvABF2 (a), VvABI5 (b) and the promoter of *VvWRKY37* in tobacco leaf, respectively. The activities of luciferase were imaged using a CCD device.


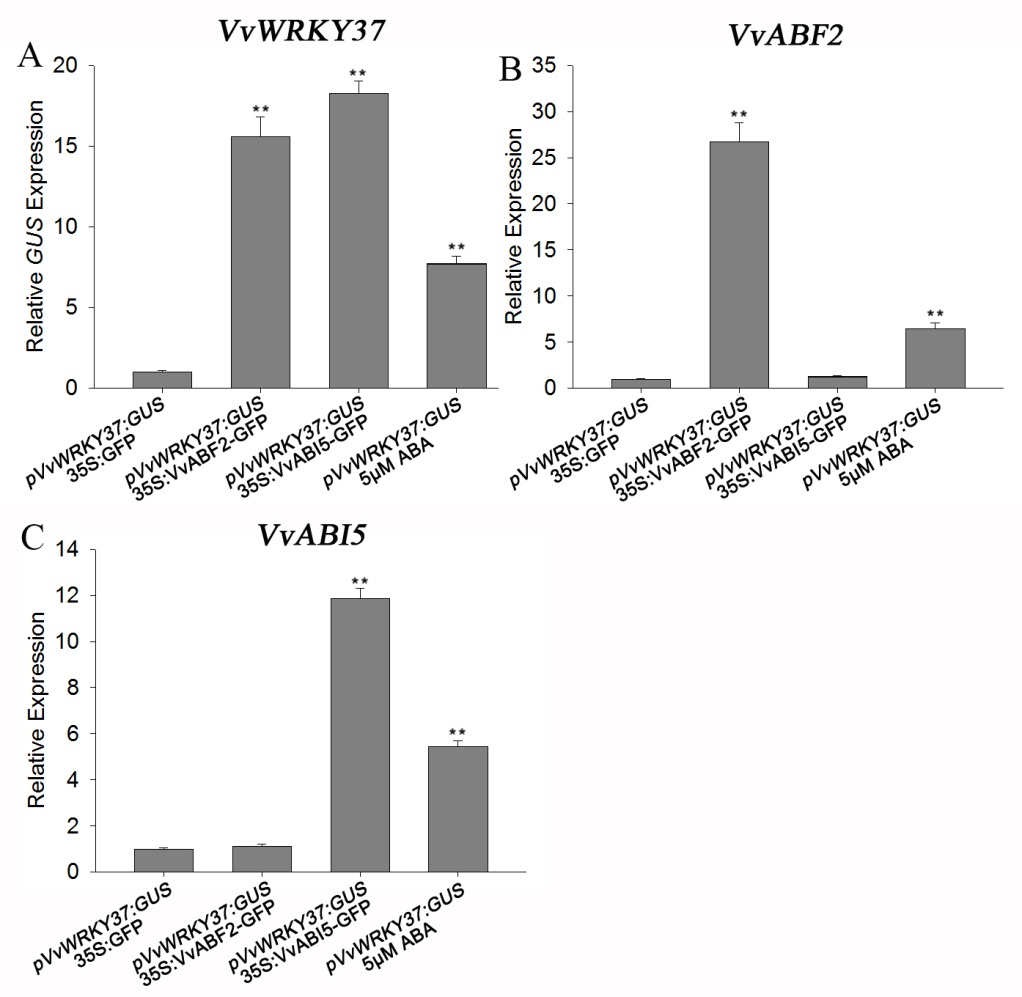


Figure S11 The transcript levels of *VvWRKY37*, *VvABF2* and *VvABI5* in transiently transformed grapevine leaves. *VvActin* serves as the reference gene. Asterisks indicate statistically significant differences as indicated: **, P < 0.01.


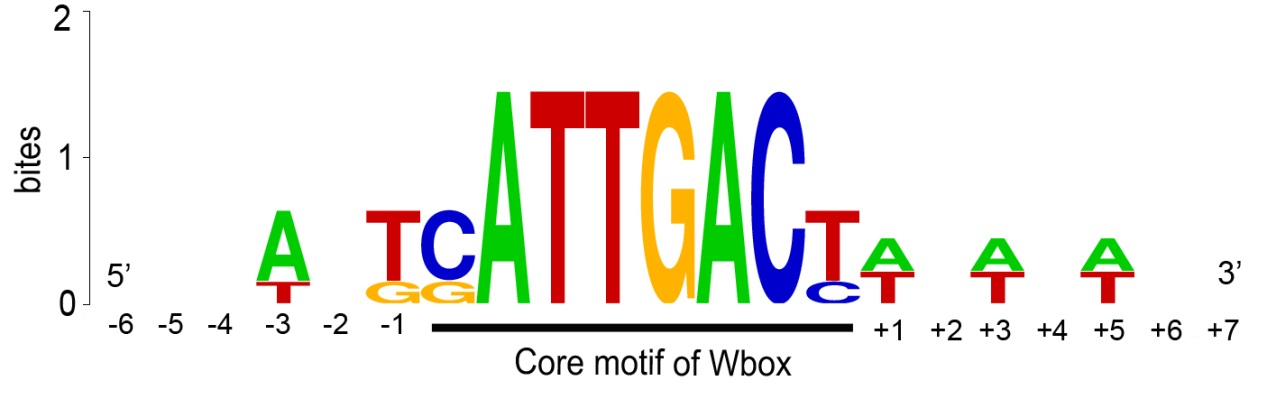


Figure S12 The oligonucleotide sequence logos of W-box element together with the flanking sequence specially bound by VvWRKY37 confirmed through EMSA assays. The core motif of W-box was highlighted with black bold underline.


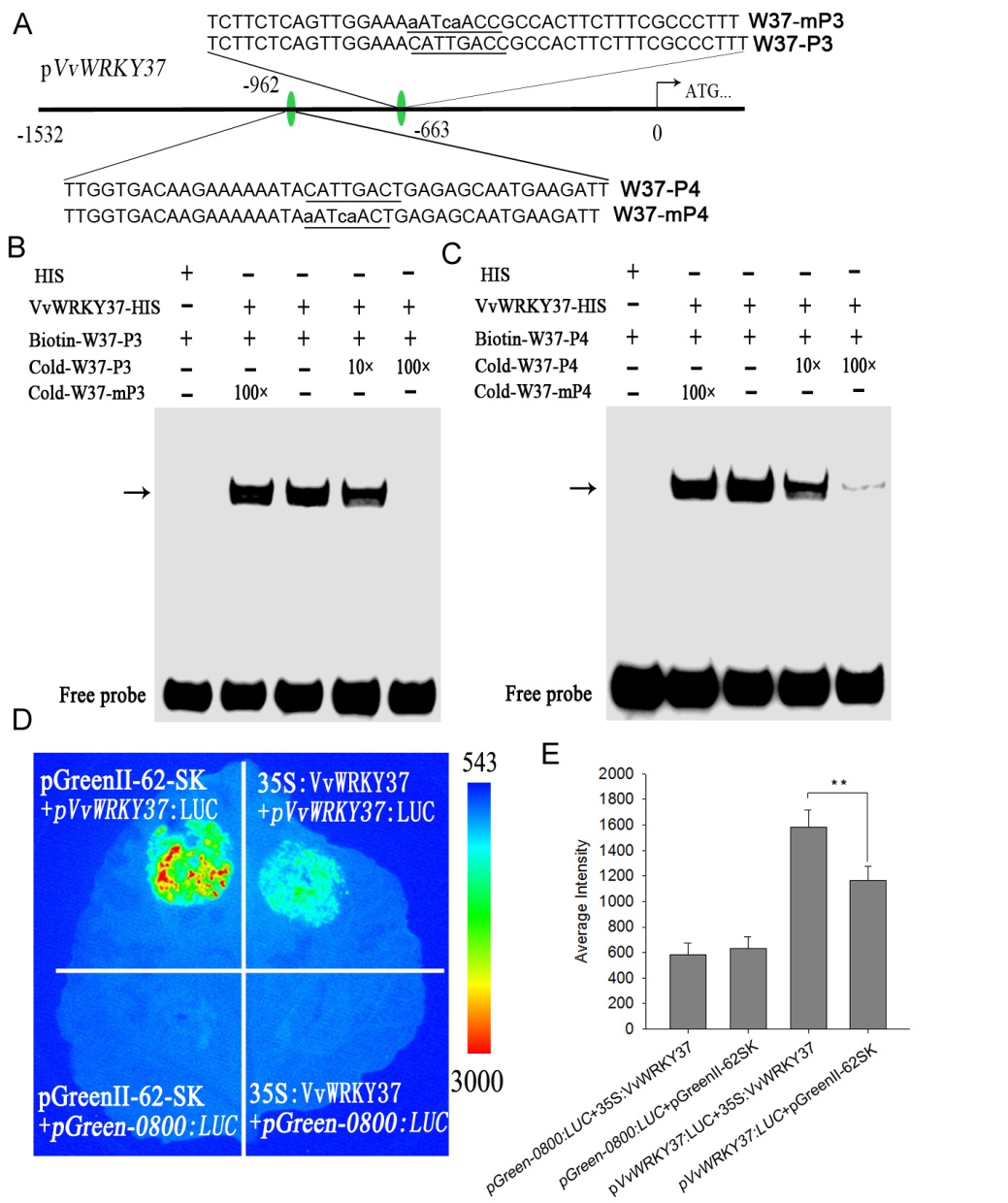


Figure S13 VvWRKY37 was a self-regulated TF through direct protein-DNA interaction. (a) The DNA probes derived from *VvWRKY37* promoter were synthesized for EMSA assays. Each probe contained a putative W-box *cis*-element 5′-CATTGACC-3′ (W37-P3) or 5′-CATTGACT-3′ (W37-P4), which was mutated into 5′-aATcaACC/T-3′. The fused protein VvWRKY37-His strongly bound W37-P3 (b) and W37-P4 (c) *in vitro*, respectively. Competition assays were performed just as described in Figure 4d. The black arrow indicated the protein-DNA complex. (d) VvWRKY37 was a self-depressed transcription factor through direct protein-DNA interplay. (e) The average intensity of fluorescence exhibited on the tobacco leaves. Each data represents the average of triple biological replicates, and error bars indicate SD. Asterisks in d indicate statistically significant differences as indicated: **, P < 0.01.
